# Hepatobiliary MRI: Signal intensity based assessment of liver function correlated to ^13^C-Methacetin breath test

**DOI:** 10.1038/s41598-018-27401-5

**Published:** 2018-06-13

**Authors:** Michael Haimerl, Ute Probst, Stefanie Poelsterl, Lukas Beyer, Claudia Fellner, Michael Selgrad, Matthias Hornung, Christian Stroszczynski, Philipp Wiggermann

**Affiliations:** 1 Department of Radiology, University Hospital Regensburg, Regensburg, Germany; 2 Department of Internal Medicine I, University Hospital Regensburg, Regensburg, Germany; 3 Department of Surgery, University Hospital Regensburg, Regensburg, Germany

## Abstract

Gadoxetic acid (Gd-EOB-DTPA) is a paramagnetic MRI contrast agent with raising popularity and has been used for evaluation of imaging-based liver function in recent years. In order to verify whether liver function as determined by real-time breath analysis using the intravenous administration of ^13^C-methacetin can be estimated quantitatively from Gd-EOB-DTPA-enhanced MRI using signal intensity (SI) values. 110 patients underwent Gd-EOB-DTPA-enhanced 3-T MRI and, for the evaluation of liver function, a ^13^C-methacetin breath test (^13^C-MBT). SI values from before (SI_pre_) and 20 min after (SI_post_) contrast media injection were acquired by T1-weighted volume-interpolated breath-hold examination (VIBE) sequences with fat suppression. The relative enhancement (RE) between the plain and contrast-enhanced SI values was calculated and evaluated in a correlation analysis of ^13^C-MBT values to SI_post_ and RE to obtain a SI-based estimation of ^13^C-MBT values. The simple regression model showed a log-linear correlation of ^13^C-MBT values with SI_post_ and RE (p < 0.001). Stratified by 3 different categories of ^13^C-MBT readouts, there was a constant significant decrease in both SI_post_ (p ≤ 0.002) and RE (p ≤ 0.033) with increasing liver disease progression as assessed by the ^13^C-MBT. Liver function as determined using real-time ^13^C-methacetin breath analysis can be estimated quantitatively from Gd-EOB-DTPA-enhanced MRI using SI-based indices.

## Introduction

To monitor patients with hepatic dysfunction, various liver function tests are used routinely in clinical practice. These tests may evaluate the increasing severity of illness, differ between stages of disease and offer a prediction of therapy outcome^1–3^. The routine part of clinical investigations is based on analysis, where the levels of non-volatile compounds, such as proteins and ions, are measured and checked for abnormalities. As a complement to blood parameters, volatile compounds carry information concerning the biochemical status of the individual, which might be examined via breath tests^4–10^.

Different orally or intravenously administered ^13^C-labeled substrates can reflect the function of specific hepatocyte compartments in real time as they are processed by liver function-dependent metabolic pathways. Therefore, cytosolic, mitochondrial and microsomal processes can be investigated non-invasively to obtain information about site-specific physiological and pathological metabolism^5^. A novel approach, which is already used in clinical routine, is the ^13^C-methacetin breath test (^13^C-MBT) established by Stockmann *et al*.^11^. The principle underlying this real-time test is the ability to metabolize ^13^C-labeled methacetin by the hepatocyte endoplasmic reticulum-located cytochrome P_450_ 1A2 (CYP1A2) into paracetamol and ^13^C-labeled formaldehyde, which will be eliminated as ^13^CO_2_^5,11–13^. After intravenous (i.v.) injection, ^13^CO_2_ will be exhaled, and a ^13^CO_2_:^12^CO_2_ ratio can be determined by a suitable device for breath analysis. Based on the values obtained by the ^13^C-MBT, real-time information of patients’ liver function can be directly obtained.

The hepatocyte-specific magnetic resonance imaging (MRI) contrast agent gadoxetic acid (Gd-EOB-DTPA) has been established and used for diagnostic purposes to detect and characterize focal liver lesions. Several studies have reported the use of Gd-EOB-DTPA-based MRI to evaluate liver function, usually expressed via the Child-Pugh score. However, no studies have yet compared the direct assessment of hepatic metabolic capacity and the indirect estimation of liver function based on MRI.

The purpose of this retrospective analysis was to evaluate the diagnostic performance of the Gd-EOB-DTPA-enhanced signal intensity (SI)-based quantification of liver function compared with an established liver function test, the ^13^C-MBT.

## Results

**Figure 1:**
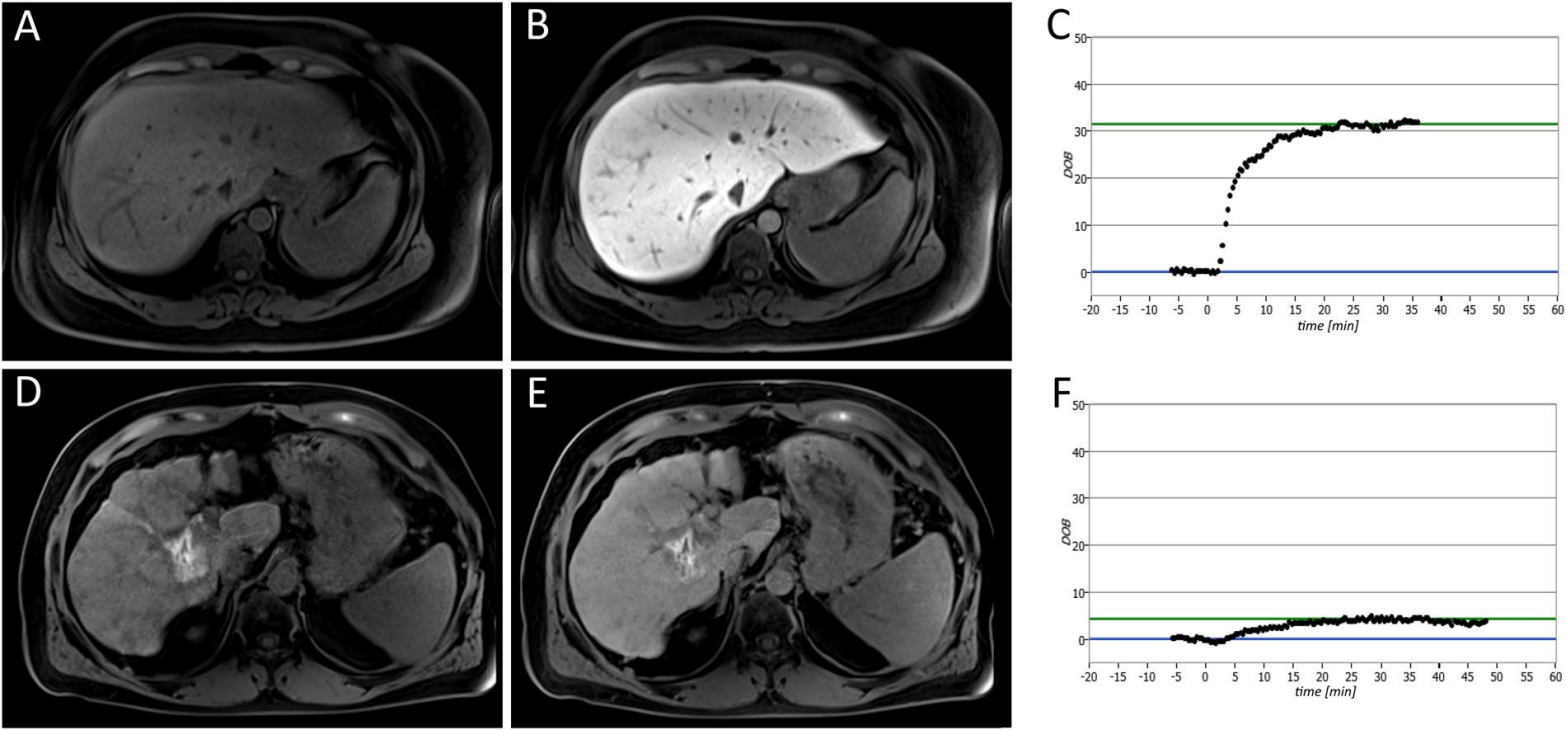
Liver function analysis in patients with normal liver function (**A**,**B**,**C**) and severely impaired liver function (**D**,**E**,**F**). The patients underwent plain (**A**,**D**) and Gd-EOB-DTPA-enhanced HBP (**B**,**E**) T1-weighted VIBE sequences with fat suppression, as well as a ^13^C-MBT, over a maximum time span of 60 min (**C**,**F**). In the case of the displayed patients, a ^13^C-MBT readout value of 409 µg/kg/h (**C**) was considered normal liver function (SI_pre_, 182.67; SI_post_, 441.67; RE, 1.42), while a ^13^C-MBT readout value of 57 µg/kg/h (**F**) was considered impaired liver function (SI_pre_, 258.50; SI_post_, 332.83; RE, 0.29). The lesion observed in the liver with impaired function (**D**,**E**) was caused by former radiofrequency ablations treatments. The delta-over-baseline (DOB) was assessed inline automatically and describes the increase in the R_PDB_-corrected ^13^CO_2_:^12^CO_2_ ratio to the basal value (blue line). The evaluated ^13^C-MBT value was calculated as the product of the DOB_max_, R_PDB_, CO_2_ production and molar mass of ^13^C-methacetin per body weight^13^. The DOB_max_ (green line) was defined after an increase in DOB was no longer observable. At the time point 0, the ^13^C-methacetin was applied via bolus injection.

**Figure 2:**
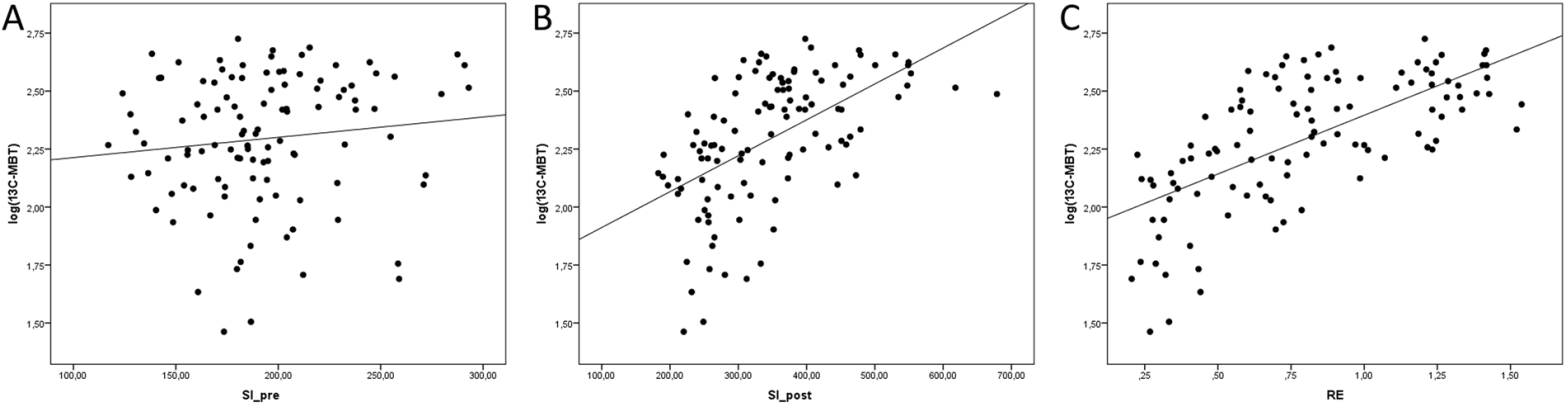
Correlation analysis of SI-based liver function indices to logarithmic values of ^13^C-MBT readout in scatterplots. The SI values obtained without contrast enhancement (SI_pre) show no predictive power for the logarithmic values of ^13^C-MBT (r = 0.213, p = 0.120; **A**), while the contrast-enhanced SI values (SI_post) show a significant correlation (r = 0.554, p < 0.001; **B**). A strong linear prediction of logarithmic ^13^C-MBT values can be observed for the relative enhancement (RE) values (r = 0.665, p < 0.001; **C**).

The baseline characteristics of the 110 patients (83 men and 27 women; mean age, 60.84 ± 9.52 years; range, 39–82 years) who were included in this study and underwent a ^13^C-MBT and T1-weighted VIBE MRI are summarized in Table 1. Representative T1-weighted MRI scans and the corresponding SI-based indices, SI_pre_, SI_post_ and RE, are shown in Figs 1 and 2.

**Table 1:** Characteristics for all patients and each ^13^C-MBT readout category.

parameters	n = 110	^13^C-MBT categories		
		1 >315.0 [µg/kg/h] n = 33	2 140.0–315.0 [µg/kg/h] n = 46	3 <140.0 [µg/kg/h] n = 31
male	83 (75.5%)	14 (42.4%)	43 (93.5%)	26 (83.9%)
female	27 (24.5%)	19 (57.6%)	3 (6.5%)	5 (16.1%)
age [years]	60.84 ± 9.52	59.88 ± 11.02	62.33 ± 9.40	59.65 ± 7.81
height [cm]	172.92 ± 7.62	168.97 ± 8.62	175.37 ± 7.27	173.48 ± 5.05
weight [kg]	85.84 ± 16.03	77.74 ± 19.80	89.96 ± 12.83	88.35 ± 12.80
SI_pre_	192.98 ± 38.40	204.80 ± 41.08	185.30 ± 35.71	191.80 ± 37.42
SI_post_	347.36 ± 100.38	414.03 ± 87.85	345.59 ± 97.14	279.03 ± 67.09
RE	0.80 ± 0.37	1.04 ± 0.27	0.86 ± 0.35	0.46 ± 0.20
^13^C-MBT [µg/kg/h]	234.66 ± 124.08	391.21 ± 53.28	217.78 ± 49.39	93.03 ± 33.59

Values indicate the mean ± standard deviation. RE: relative enhancement as a function of SI-based indices. SI: mean signal intensity of the liver. ^13^C-MBT: ^13^C-labeled methacetin metabolism liver function breath test.

### SI in T1-weighted MRI scans compared with ^13^C-MBT readout

In a simple linear regression model, RE values were strong linear predictors of the logarithmic values of uncategorized ^13^C-MBT readout values (r = 0.665, p < 0.001), while the SI values obtained from the HBP showed less predictive power (r = 0.554, p < 0.001). In contrast, SI values obtained without contrast enhancement showed no significant correlation (p = 0.120) (Table 2). Scatterplots of SI_pre_, SI_post_ and RE values plotted against the logarithmic values of ^13^C-MBT are shown in Fig. 2.

**Table 2:** Simple linear regression models of SI-based indices with logarithmic values of ^13^C-MBT.

		B (95% CI)	p-value	r
Log(^13^C-MBT)	RE	0.507 (0.398–0.615)	<0.001	0.665
	SI_pre_	0.001 (−0.001–0.002)	0.213	0.120
	SI_post_	0.002 (0.001–0.002)	<0.001	0.554

RE: relative enhancement as a function of SI-based indices. SI: mean signal intensity of the liver. ^13^C-MBT: ^13^C-labeled methacetin metabolism liver function breath test. r: correlation coefficient. B: linear regression coefficient. CI: confidence interval.

### SI-based results compared with ^13^C-MBT readout categories

**Figure 3:**
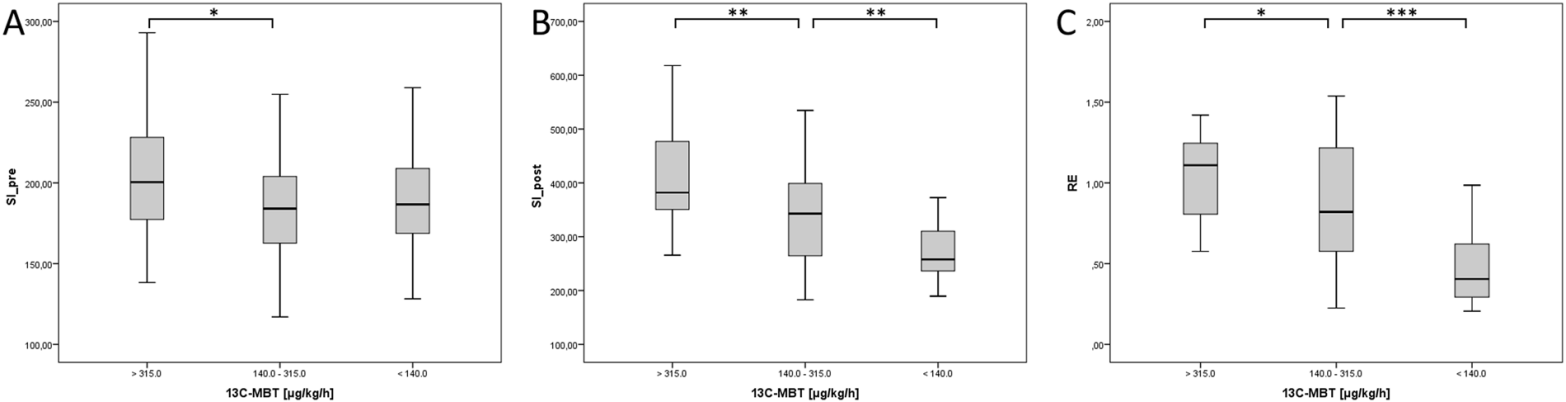
Boxplot analysis of SI-based indices separated by ^13^C-MBT readout categories. Native SI values (SI_pre; **A**) show no significant difference between the different ^13^C-MBT readout categories, except for Categories 1 and 2. SI values obtained after contrast enhancement (SI_post; **B**) and corrected by native SI values (RE; **C**) show significant differences among the ^13^C-MBT readout categories. *p < 0.05; **p < 0.01; ***p < 0.001.

Patients with normal liver function (Category 1) had a mean ^13^C-MBT readout of 391.21 ± 53.28 µg/kg/h, whereas patients with intermediate liver function (category 2) had a mean ^13^C-MBT readout value of 217.78 ± 49.39 µg/kg/h, and patients with severely impaired liver function had a mean ^13^C-MBT readout value of 93.03 ± 33.59 µg/kg/h (Table 1). All pairwise comparisons between Categories 1, 2 and 3 showed significant differences (p ≤ 0.05) in the evaluated SI-based indices, except for SI_pre_ values between Categories 1 and 3 and Categories 2 and 3 (Fig. 3A). The SI values obtained from the HBP (SI_post_; p < 0.005; Fig. 3B) and the RE values (p < 0.05; Fig. 3C) differed significantly among the three ^13^C-MBT based categories.

## Discussion

One of the most common types of liver cancer is HCC, with a global cancer mortality rate of 9.1%^14^. As HCC is mostly based on preexisting liver cirrhosis, the early detection and assessment of liver cirrhosis is of high clinical relevance. Patients benefit from rapid and accurate examinations of the liver condition. Thus, the ^13^C-MBT has become more common, as it can provide liver failure predictions, liver transplant control, and liver disease severity estimations^13,15–17^. The test is based on the analysis of volatile components exhaled during the ^13^C-methacetin metabolism by CYP1A2 in the endoplasmic reticulum^12^. Different studies have demonstrated the predictive power of enzymatic hepatocyte functionality for liver resection^13,15,17–21^. Even in cases of non-cirrhotic, early-stage and primary biliary cirrhosis, the ^13^C-MBT can reliably indicate decreased liver function^10^. Nevertheless, this tool has some diagnostic restrictions, as it is unable to determine and distinguish between areas of reduced hepatocyte function and healthy areas. Therefore, ^13^C-MBT values describe the function of the whole liver. However, diagnostic imaging techniques might provide these details, which are crucial for both liver resection and liver transplantation. To reduce the number of different examinations, researchers aim to establish liver tests capable of not only reliably reflecting liver function but also revealing hepatic lesions in a single examination.

To this end, Gd-EOB-DTPA-enhanced MRI has been established as a promising approach for assessing liver function in the past few years. Gd-EOB-DTPA consists of a gadolinium ion covalently bound to a lipophilic ethoxybenzyl group and is absorbed by intact hepatocytes via triggered ATP-dependent organic anion-transporting polypeptide (OATP1 B1/B3) channels. Subsequently, Gd-EOB-DTPA will be excreted by multidrug-resistance protein 2 (MRP2) into the biliary system^22–24^. The strength of Gd-EOB-DTPA is its hepatocyte-specific character, which improves the contrast-to-noise ratio, as it will not be taken up by cells other than hepatocytes (e.g., cells in metastatic lesions)^25–27^. At 20 min after contrast agent application, a significant increase in the SI of the liver parenchyma is recorded, and the extent of this phenomenon is dependent on the integrity of the liver parenchyma. The highest SI is exhibited by healthy liver tissue, while cirrhotic liver parenchyma shows only a slight increase in SI^28,29^. A loss of functioning hepatocytes, bridging of portal spaces or nodular regeneration of the liver parenchyma is associated with liver cirrhosis and hindered hepatocyte contrast agent uptake, causing decreased SI-based values^30^.

The estimation of SI-based indices provides insights into regional hepatocyte-specific function, efficiency, functionality and condition^31^. Nevertheless, the analysis is restricted by the relative character of the obtained SI values^32^. However, by the mathematical computation of SI_post_ with SI_pre_, a liver function index with increased reliability can be achieved. By calculating the RE, we correct the enhanced SI values to gain a SI ratio independent from artificial signal enhancement with increased explanatory power^29,33–38^.

To the best of our knowledge, this is the first study comparing SI-based MRI values reflecting hepatocyte OATP1 B1/B3 and MRP2 pathway activity with the ^13^C-MBT.

We could demonstrate that the SI-based MRI values assessed in the HBP reflect liver function in a suitable manner, as estimated by ^13^C-MBT readout values. Similar to a previous study^39^, we observed that patients with normal liver function expressed the highest SI-based values (Category 1: SI_pre_, 204.80 ± 41.08; SI_post_, 414.03 ± 87.85; RE, 1.04 ± 0.27), while patients with decreased liver function showed decreased SI-based values (Category 2: SI_pre_, 185.30 ± 35.71; SI_post_, 345.59 ± 97.14, RE, 0.86 ± 0.35; Category 3: SI_pre_, 191.80 ± 37.42; SI_post_, 279.03 ± 67.09; RE, 0.46 ± 0.20). In a correlation analysis of SI-based indices to ^13^C-MBT values, we were able to show that SI-based RE values significantly support the ^13^C-MBT findings (RE, r = 0.665, p < 0.001) and therefore reflect liver functionality. Similar to Utsunomiya *et al*.^31^, in this study, we tested the correlation of the mean SI obtained after contrast agent application (SI_post_) to the results of a liver function test. In their case, the tested SI values showed a higher prediction of indocyanine green dye (ICG) retention at 15 min (r = −0.67, p < 0.01) than we obtained when testing against ^13^C-MBT values (r = 0.554, p < 0.001). However, this difference seems plausible because the ICG clearance test is, similar to the GD-EOB-DTPA pathway, dependent from OATP transporter activity. Therefore, we expected a slightly less pronounced correlation between the ^13^C-MBT and SI-based values, as the ^13^C-MBT relies on an enzymatic metabolism, whereas contrast-enhanced MRI relies on OATP channel-triggered contrast agent uptake. In general, the ICG clearance test has certain limitations, as constant hemodynamic conditions (stable liver perfusion rate and hepatic blood flow) are required for liver function analysis^40–42^ and, in cases of cholestasis and hyperbilirubinemia, carrier competition of bilirubin and ICG at the OATP1 transporter might occur^43,44^. It is also known that various drugs (e.g., rifampicin) exert inhibitory effects on the OATP pathway and might influence hepatocyte ICG uptake^45,46^. Similar to the findings of Tamada *et al*.^36^, we could show that the RE of the liver parenchyma serves as a reliable tool for liver function classification, as the RE significantly differs among different stages of liver function (p ≤ 0.02). Additionally, it has been shown that the hepatic enhancement during Gd-EOB-DTPA-enhanced MRI is strongly affected by the degree of liver cirrhosis, as expressed by the ICG test, the Child-Pugh score or the MELD score^34,47^. These studies have shown that Gd-EOB-DTPA-enhanced MRI has potential as a reliable tool for liver function estimation in addition to its already established implementation for hepatic lesion detection.

Our study has several limitations. First, ROI placement may cause some variations due to the possible nonhomogeneous distribution of parenchymal changes. However, using the average of six repeated ROI measurements across an area of the liver parenchyma should provide reliable values. Second, this study was retrospective in nature, with only a limited patient population. Third, the lack of histopathology is another potential limitation.

In conclusion, SI-based indices, such as the RE and contrast-enhanced SI values, can be used to determine liver function as assessed by ^13^C-MBT.

## Materials and Methods

### Patients

Local institutional review board approval of the University Hospital Regensburg was obtained for ^13^C-MBT and Gd-EOB-DTPA-enhanced MRI at 3 T. Only data from written informed consent patients were included for this analysis, also the study was performed in accordance with the relevant guidelines and regulations.

The retrospective analysis includes 110 patients (83 men and 27 women; median age, 61 years) who underwent both a ^13^C-MBT and Gd-EOB-DTPA-enhanced MRI at 3 T. The patients underwent Gd-EOB-DTPA-enhanced T1-weighted volume-interpolated breath-hold examination (VIBE) MRI sequences with fat suppression. The included patients did not have known reactivity to liver-specific MRI contrast media, ^13^C-methacetin intolerance or renal-specific contraindications to either MRI and Gd-EOB-DTPA administration.

The patients underwent MRI and a liver function test for the following reasons:

- active hepatocellular carcinoma monitoring in the case of no (n = 1) or known liver cirrhosis (n = 30)
- follow-up in the case of known secondary malignancy (cholangiocarcinoma, n = 3; duodenal carcinoma, n = 1; rectal cancer, n = 4; sigma carcinoma, n = 2; uveal melanoma, n = 1) or benign hepatic lesion (focal nodular hyperplasia (FNH), n = 1; hemangioma, n = 3)
- preinterventional assessment in the case of known hepatocellular carcinoma (HCC) (n = 6) or known secondary liver malignancy / focal hepatic lesion (cholangiocarcinoma, n = 2; hemangioma, n = 1; mamma carcinoma, n = 1; rectal cancer, n = 4; sigma carcinoma, n = 1)
- postinterventional assessment in the case of known HCC (n = 19) or known secondary liver malignancy (cholangiocarcinoma, n = 3; colon carcinoma, n = 1; mamma carcinoma, n = 1; rectal cancer, n = 1; sigma carcinoma, n = 2; thymoma, n = 1)
- in the case of suspected liver disease or focal hepatic lesions with known cirrhosis (n = 12), overlap syndrome (n = 1), Budd-Chiari syndrome (n = 1), cholangiocarcinoma (n = 2), carcinoid of the ileum (n = 1), rectal cancer (n = 3), or sigma carcinoma (n = 1)

The main reasons for liver cirrhosis were alcoholic steatohepatitis (n = 30), hepatitis B infection (n = 12) and hepatitis C infection (n = 13). Only 2 patients suffered from non-alcoholic steatohepatitis (NASH). Detailed insights in underlying disease can be seen in Table 3.

**Table 3:** Underlying diseases for MRI examination and ^13^C-MBT for each medical case.

		patients (n = 110)
HCC	with cirrhosis	62
	without cirrhosis	1
liver disease	liver cirrhosis	5
autoimmune disease	overlap syndrome (AIH and PBC)	1
benign liver lesion	focal nodular hyperplasia (FNH)	1
	hemangioma	4
hepatic vein thrombosis	Budd-Chiari syndrome	1
secondary liver malignancies	carcinoma of the ileum or duodenum	2
	cholangiocarcinoma	10
	colon carcinoma	1
	mamma carcinoma	2
	rectal cancer	12
	sigma carcinoma	6
	thymoma	1
	uveal melanoma	1

### ^13^C-MBT

The ^13^C-MBT was performed 24 h before or after the MRI scan, according to published recommendations^11,13^. The patients fasted for at least 3 hours before the ^13^C-MBT. Ten minutes before the i.v. injection of ^13^C-methacetin, a breath ^13^CO_2_:^12^CO_2_ ratio control was recorded to calculate the delta-over-baseline (DOB). Then, 2 mg/kg body weight ^13^C-methacetin was injected via i.v. bolus and flushed with 20 mL of 0.9% sodium chloride. The volatile analysis, performed by modified nondispersive isotope-selective infrared spectroscopy (FANci2-db16, Fischer Analysen Instrumente, Leipzig, Germany), was started immediately after the injection to track the hepatocyte-specific enzymatic ^13^CO_2_ production.

For the statistical analysis, the patients were grouped according to their ^13^C-MBT readout into 3 categories: patients with normal liver function (Category 1): ^13^C-MBT > 315 [µg/kg/h]; patients with intermediate liver function (Category 2): ^13^C-MBT 315–140 [µg/kg/h]; and patients with severely impaired liver function (Category 3): ^13^C-MBT < 140 [µg/kg/h]^13,16^.

### MRI

All imaging was performed using a clinical whole-body 3-T system (MAGNETOM Skyra, Siemens Healthcare, Erlangen, Germany). T1-weighted VIBE sequences with fat suppression (repetition time (TR), 3.09 ms; echo time (TE), 1.17 ms, 2.49 ms; flip angle, 10°; parallel imaging factor, 2; slices, 64; reconstructed voxel size, 1.25 × 1.25 × 3.0 mm^3^; measured voxel size, 1.71 × 1.25 × 4.5 mm^3^; acquisition time, 14 sec) were acquired during breath-holding before and 20 min after Gd-EOB-DTPA (Primovist®, Bayer Healthcare, Berlin) administration. Every sequence covered the entire liver before Gd-EOB-DTPA administration and in the hepatobiliary phase (HBP) after 20 min.

The patients received a Gd-EOB-DTPA dose (0.025 mmol/kg body weight) adapted to their body weight administered via bolus injection at a flow rate of 1 mL/s, followed by 20 mL of 0.9% sodium chloride.

### Image analysis

**Figure 4:**
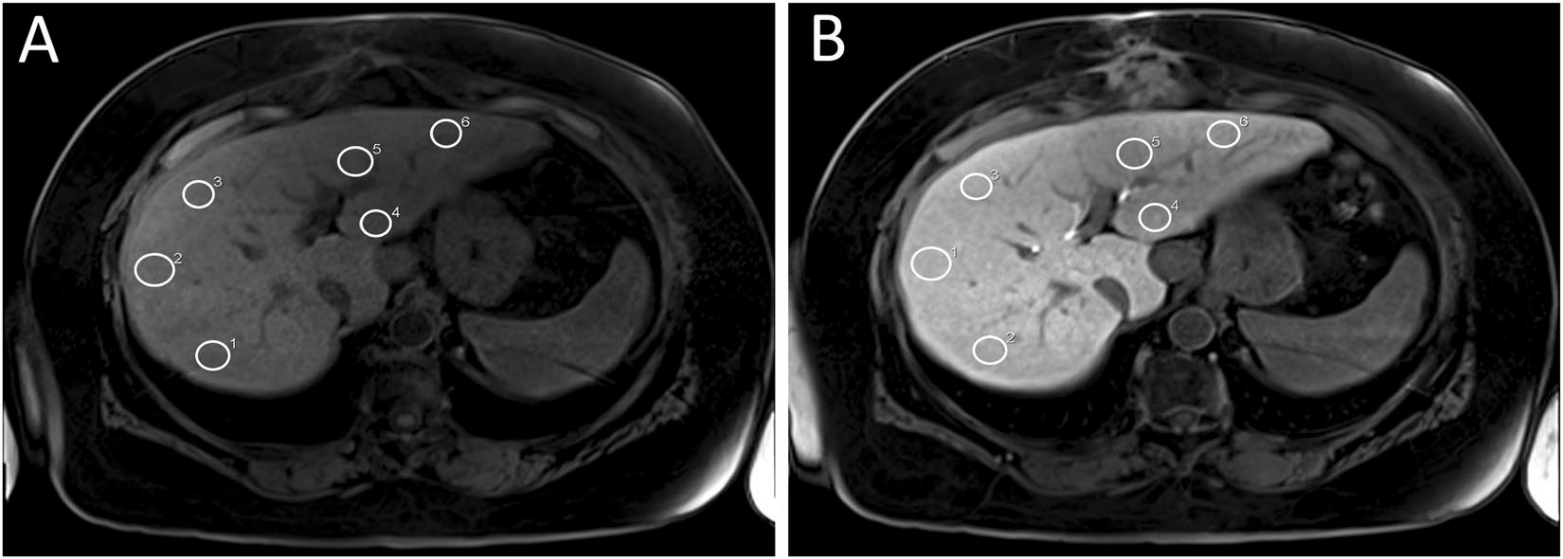
Representative example of ROI placement in unenhanced (**A**) and Gd-EOB-DTPA-enhanced VIBE (**B**) scans of a patient with normal liver function. White circles mark ROIs manually placed at identical places in the right and left lobe of the liver.

Operator-defined region-of-interest (ROI) measurements were used to obtain the mean SI values from the T1-weighted VIBE images (before and after Gd-EOB-DTPA injection). ROIs were manually placed at identical locations in every sequence, avoiding liver lesions, major branches of the portal and hepatic veins, and imaging artifacts. In total, 6 ROIs (3 each in the right and left lobes) were defined in the VIBE images (Fig. 4). Each ROI was a circle that was made as large as possible (liver parenchyma: 1.1 cm^2^–4.6 cm^2^) and manually adjusted between sequences if necessary.

The relative enhancement (RE) of the liver was calculated according to following formula:

1 $${\rm{RE}}=\,\frac{{\rm{SI}}\,\mathrm{post}\,-\,\mathrm{SI}\,{\rm{pre}}}{{\rm{SI}}\,{\rm{pre}}}$$

### Statistical analysis

The different ^13^C-MBT readout categories were compared as non-parametric independent samples by the Mann-Whitney-U test. The predictive power of SI-based indices was determined by simple linear regression models, and the optimal curve fit was assessed visually. In all tests, the statistical significance level was set to 0.05 (two-sided). All analyses were performed using SPSS software (version 24; IBM, Chicago, IL, USA).

### Data availability

All data that support the findings of this study are provided in the manuscript. Raw data used in this work are available on reasonable request.
